# Fenamates as Potential Therapeutics for Neurodegenerative Disorders

**DOI:** 10.3390/cells10030702

**Published:** 2021-03-22

**Authors:** Jaunetta Hill, Nasser H. Zawia

**Affiliations:** 1 Department of Biomedical and Pharmaceutical Sciences, University of Rhode Island, Kingston, RI 02881, USA; jaunettahill@uri.edu; 2 Interdisciplinary Neuroscience Program, University of Rhode Island, Kingston, RI 02881, USA; 3 George and Anne Ryan Institute for Neuroscience, University of Rhode Island, Kingston, RI 02881, USA

**Keywords:** tolfenamic acid, mefenamic acid, flufenamic acid, meclofenamic acid, fenamate, NSAID, neurodegenerative, Alzheimer’s disease

## Abstract

Neurodegenerative disorders are desperately lacking treatment options. It is imperative that drug repurposing be considered in the fight against neurodegenerative diseases. Fenamates have been studied for efficacy in treating several neurodegenerative diseases. The purpose of this review is to comprehensively present the past and current research on fenamates in the context of neurodegenerative diseases with a special emphasis on tolfenamic acid and Alzheimer’s disease. Furthermore, this review discusses the major molecular pathways modulated by fenamates.

## 1. Introduction

Nonsteroidal anti-inflammatory drugs (NSAIDs) are commonly prescribed therapeutics, such as ibuprofen and aspirin, that are well-documented for exerting anti-inflammatory, analgesic, and antipyretic effects [1]. These effects are due to the ability of NSAIDs to inhibit cyclooxygenases (COX), which prevents the conversion of arachidonic acid to eicosanoids resulting in a reduction of proinflammatory prostaglandin (PG) synthesis [2,3]. Thus, NSAIDs have been prescribed to treat a variety of illnesses including migraines, pain, arthritis, fever, and blood clots.

The first NSAID, acetylsalicylic acid or aspirin, was synthesized in 1897 by Felix Hoffmann [3]. The mechanistic pathway of NSAIDs was not fully understood until the early 1990s, approximately 90 years after the production of aspirin when COX enzymes and PGs were characterized [4]. COX enzymes are found in the cardiovascular, neuronal, renal, immune, gastrointestinal, and reproductive systems in the human body [4]. There are two well-characterized cyclooxygenase isoenzymes known as COX-1 and COX-2. COX-1, the constitutive isoform, produces PGs, which are involved in cellular “housekeeping” and gastrointestinal protection [5]. COX-2 is constitutively expressed in the kidney and brain and is inducible in cell types such as macrophages and colorectal cancer cells [6]. It can also be increased by cytokines, growth factors, and other inflammatory stimuli [7]. COX-1 inhibition in the gastrointestinal tract causes a reduction in prostaglandin secretion and its cytoprotective effect in the gastric mucosa, which may lead to the development of gastrointestinal ulcers, and thus COX-2 inhibitors were originally thought to be safer [8]. All NSAIDs inhibit both COX-1 and COX-2 in vitro, as they are nonselective. Generally, the ratio between the 50% inhibitory concentration (IC_50_) values for COX-1 and COX-2 have been used to determine the selectivity of the therapeutics [6]. However, these criteria have been described as arbitrary terms to characterize “selective” vs. “nonselective” inhibitors [6]. Notably, in 2004, Merck and Co announced the voluntary withdrawal of a popular COX-2 inhibitor, rofecoxib (Vioxx), worldwide after substantial evidence from the VIGOR study and several other large randomized controlled clinical trials proving increased risk of cardiovascular events following usage of the drug [5,9].

**Figure 1:**
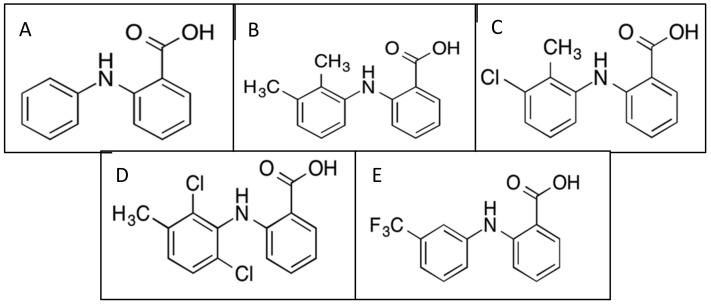
Fenamate chemical structures: (**A**) fenamic acid parent structure; (**B**) mefenamic acid structure; (**C**) tolfenamic acid structure; (**D**) meclofenamic acid structure; (**E**) flufenamic acid structure.

Fenamates are a subgroup of NSAIDs, derived from a fenamic acid core structure, which includes several major clinically prescribed drugs such as mefenamic acid (MFA), tolfenamic acid (TA), meclofenamic acid (MCFA), and flufenamic acid (FFA) (Figure 1). Fenamates are carboxylic acids with pKa values of approximately 4 and are >99% ionized at physiological pH levels [10]. They are eliminated mainly by hydroxylation and glucuronidation and excreted in the urine [10]. MFA is the most potent inhibitor of COX-2, and TA inhibits both COX-1 and COX-2 [11]. All four fenamates have been studied for various clinical applications outside of pain relief, including cancer, stroke, Alzheimer’s disease, Huntington’s disease, and epilepsy.

Fenamates have been implicated as therapeutic for many diseases and ailments outside of pain management. Research has suggested that fenamates may be great candidates for drug repurposing because of the wide range of therapeutic benefits. This review focuses on the multiple atypical uses of fenamates and the mechanistic disease pathways that may be affected.

The following paper summarizes the available data on the clinically relevant fenamates, their potential neuro-clinical impact, and their mechanisms of action, with a special emphasis on TA and neurodegenerative diseases.

## 2. Fenamate Pharmacokinetics and Pharmacodynamics

Pharmacodynamically, all fenamates exhibit anti-inflammatory, analgesic, and anti-pyretic activity through COX inhibition in animal models and humans [12].

### 2.1. Tolfenamic Acid

Tolfenamic acid or TA is available as Clotam Rapid in Europe, where it is commonly prescribed to treat migraine headaches and in veterinary care. Typically, 200 mg is prescribed as the dosage for migraine treatment. TA has an oral absorption with a mean lag time of 32 min and a peak plasma concentration of 11.1 mcg/mL with bioavailability around 60–75% [13]. TA has a high protein-binding distribution of approximately 99.7% of the administered dose, with peak plasma concentrations being achieved in 1–2 h after oral administration [13]. The half-life is 8.1–13.5 h if orally administered and 6.1 h if taken intravenously, and first-pass metabolism accounts for 20% of the administered dose and it is eliminated relatively fast hepatically. The LD_50_ is 225 mg/kg in rats following oral administration [13]. TA was confirmed to cross the blood–brain barrier after intravenous administration in guinea pigs [14]. After administration of 100 mg/kg TA, mouse serum obtained a concentration of 10.85 µg/mL and the concentration in the brain was 1.21 µg/g [14].

### 2.2. Mefenamic Acid

Mefenamic acid or MFA is available in the USA as Ponstel and is prescribed to treat mild to moderate pain, and is rapidly absorbed following oral administration with a mean absorption of 30.5 mcg/h/mL with a single oral dose of 500 mg [15]. The typical dose is 500 mg, and the elimination half-life is approximately 2 h, with peak plasma levels observed at 2–4 h [15]. Typically, greater than 90% is found bound to albumin and it is expected to be excreted in human breast milk [15]. MFA is metabolized by cytochrome p450 enzyme CYP2C9 and approximately 52% of mefenamic acid is excreted into the urine [15]. Fecal elimination accounts for 20% of the dose mainly in the form of unconjugated 3-carboxymefenamic acid [15]. MFA is a competitive, time-dependent, and reversible inhibitor for both COX-1 and COX-2, and up to 80% of oral ingestion typically absorbed [16].

### 2.3. Meclofenamic Acid

Meclofenamic acid or MCFA was previously prescribed in 100 mg doses to treat mild to moderate pain and idiopathic dysmenorrhea in the USA. MCFA is rapidly absorbed with peak plasma concentrations at 0.5 to 2 h [17]. The elimination half-life ranges from 0.8 to 2.1 h [17]. MCFA is >99% bound to plasma proteins [17]. Approximately 30–62% is excreted in the urine and the remaining percent excreted in the feces [17]. The LD_50_ is between 100 and 109 mg/kg following oral or intraperitoneal in rats [17].

### 2.4. Flufenamic Acid

Flufenamic acid or FFA is typically prescribed in 200 mg doses per 8 h to treat rheumatic disorders, pain, and inflammation in several countries such as Japan, Switzerland, and Taiwan. The LD_50_ is 249 mg/kg following oral administration to rats [17]. Other pharmacokinetic information was not readily available for this drug.

### 2.5. Adverse Drug Reactions

Approximately 40 years after Felix Hoffman synthesized acetylsalicylic acid, the evidence demonstrated that aspirin could cause gastrointestinal injury due to its acidic properties [18]. The risk of gastrointestinal injury can also be attributed to the fact that COX-1 plays an important protective role in the gut by stimulating the synthesis and secretion of mucus and bicarbonate, which increases mucosal blood flow and promotes epithelial proliferation [18]. NSAIDs also increase the risk of kidney damage and nephrological complications and may cause hepatotoxicity due to prostaglandin H synthase (PHGS)-derived prostanoid inhibition [12]. Hepatotoxicity due to either hypersensitivity or metabolic aberration is another complication of NSAID use; however, it is less common than renal cardiovascular and gastrointestinal damage [12]. Cardiovascular events are the most common adverse reaction to NSAIDs, and the mechanism by which certain COX-2 inhibitors contribute to cardiovascular events is likely due to disbalance between thrombogenic and anti-aggregatory prostanoids, and as a result, PGI-2 and prostacyclin formation decreases [19,20,21].

## 3. Fenamates and Alzheimer’s Disease (AD)

Epidemiological studies have shown that a very low frequency of Alzheimer’s disease (AD) is reported in patients who have rheumatoid arthritis or leprosy and are treated with NSAIDs for a prolonged period [22]. It has even been suggested that NSAID use decreases the risk of developing AD, improves cognitive deficits, and slows the decline in patients with AD [23,24]. However, there has been a great amount of controversy over the efficacy of NSAID use to prevent AD-related cognitive impairment and biomarkers [25]. A meta-analysis provided evidence that NSAID use may result in a 20% risk reduction for AD [26]. Alternatively, the INTREPAD 2-year randomized placebo-controlled trial recruited cognitively healthy individuals who were deemed at risk of developing dementia and the trial concluded that the use of naproxen did not affect cognition, magnetic resonance imaging (MRI), or cerebral spinal fluid (CSF) biomarkers [25]. Fenamate NSAIDs have been overlooked in the majority of clinical trials and epidemiological studies. This section summarizes the available recent literature on the efficacy of fenamate NSAID use on AD-related biomarkers and cognitive deficits.

AD is characterized by the presence of cortical intracellular tau tangles and extracellular beta-amyloid plaques and is the most common form of dementia affecting over 5 million Americans and 14 million people worldwide [27]. There are two forms of AD associated with onset age. Late-onset AD (LOAD) is the most common form with an onset age of approximately 65 years. LOAD is considered sporadic—however, there have been risk factors identified with increased incidence such as the APOE ε4 gene, environmental toxicants, high-fat diet, and lack of exercise [28]. Early-onset AD (EOAD) accounts for approximately 5–6% of all AD cases and is associated with a rare inherited genetic disposition to develop AD at a young age [29]. The onset age for EOAD is approximately 30–50 years old.

Currently, there are few drugs approved to treat AD symptoms but there are no Federal Drug Administration (FDA)-approved drugs that are considered disease-modifying. The commonly prescribed drugs fall into two classes: cholinesterase inhibitors and N-methyl-D-aspartic acid (NMDA) receptor antagonists [29]. These therapeutics offer temporary symptomatic relief but are not considered disease-modifying. Cholinesterase inhibitors prevent the catalytic breakdown of acetylcholine, an important neurochemical for memory and learning [29]. Donepezil, galantamine, and rivastigmine are three well-known cholinesterase inhibitors. NMDA receptor antagonists, such as memantine, aid in regulating glutamate activity and are typically prescribed for moderate to severe cases of AD [29]. While there are few drug options for individuals affected by AD, disease-modifying treatment options for AD patients are critical as the global population increases and the projected cases of AD are expected to triple by 2050 [30].

Amyloid precursor protein (APP) is a type I transmembrane protein that may have a role in neurite outgrowth, synaptogenesis, neuronal protein trafficking, transmembrane signal transduction, cell adhesion, and calcium metabolism [31,32]. APP cleavage by the β-secretase, BACE-1, and subsequently by γ-secretase generates Aβ, which forms a neurotoxic oligomer that aggregates into extracellular plaques in AD [29]. Of note, BACE-1 activity is considered the rate-limiting factor in Aβ production from APP [31].

TA has been extensively studied for several potential disease-modifying effects in AD models. To our knowledge, TA treatment was first reported to decrease AD-related biomarkers in 2011 when researchers found that both 10 and 50 mg/kg of TA every other day for five weeks significantly reduced APP protein levels in the cerebral cortices of female and male C57Bl/6 mice [33]. The proposed mechanism by which TA lowered APP was via specificity protein 1 (SP1) transcription factor degradation [33,34]. Additionally, TA treatment lowered Aβ (1–42) levels in the cerebral cortices of treated guinea pigs, although not significantly. Another study reported that TA-treated hemizygous R1.40 mice administered 5 or 50 mg/kg/day resulted in a reduction of cortical APP protein and mRNA and reversed cognitive deficits as measured by the Morris water maze (MWM) and the Y-maze [35]. In the same study, TA lowered SP1 protein levels and soluble and insoluble Aβ_1–40_ and Aβ_1–42_ [35]. In differentiated SH-SY5Y human neuroblastoma cells, TA treatment lowered SP1 protein levels, reduced APP gene expression, and lowered lead-induced Aβ_40_ [36]. Adwan et al. (2014), also reported that TA treatment lowered BACE-1 gene expression and activity level in the cerebral cortices of APP YAC transgenic mice. Furthermore, human neuroblastoma cells treated with TA had significantly decreased protein levels of APP [37].

Currently, the hypothesis associated with TA treatment and its efficacy as an AD treatment revolves around SP1 degradation [34]. SP1 is an upstream zinc-finger transcription factor that regulates transcription of APP, microtubule-associated protein tau (MAPT), and cyclin-dependent kinase-5 (CDK5) activator genes and it is elevated in the frontal cortex of AD patients and the brains of AD transgenic mice [38]. In fact, silencing the Sp1 gene by small interfering RNA (siRNA) resulted in a 75% decrease in the responsiveness of the human APP promoter and SP1 and Aβ being co-localized in brains of AD patients [39,40]. The SP1 hypothesis aims to intercept upstream AD pathogenesis to result in a downstream decrease of key biomarkers: Aβ, tau, CDK5, and BACE-1. Initially, TA was studied for amyloid pathology effectiveness. However, recently, TA has been proven to be effective on tau pathogenesis, in vitro and in vivo, which further recapitulates the SP1 hypothesis [37,41,42].

The amyloid theory of AD has faced controversy in recent years. Although amyloid appears necessary for cognitive decline, it does not seem to be sufficient, nor does amyloid accumulation correlate well with the degree of cognitive decline [29,43]. However, there is sufficient evidence proving that amyloid pathology may be required for tau pathology progression in AD, including studies that have shown that tau pathology generally does not progress from the entorhinal cortex into the neocortex in the absence of co-occurring amyloid pathology [29,44]. The *MAPT* gene was first isolated and characterized in 1975 and, normally, tau has important roles in microtubule assembly, microtubule stability, and regulation of axonal transport; however, when tau is hyperphosphorylated, it loses affinity for microtubules and eventually aggregates, forming intracellular tau tangles [45,46]. The hyperphosphorylation of tau is mediated by several kinases including CDK5 and glycogen synthase kinase-3 beta (GSK3β) [47].

In 2015, one study reported that short-term TA treatment resulted in significantly reduced tau and CDK5 gene and total protein levels and gene expression in APP YAC mice [38]. Additionally, TA has significantly decreased total tau and phosphotau (ptau), sites Thr231 and Thr181, protein levels in human tau (hTau) transgenic mice specifically in the CA3 hippocampal region [42]. Recently, two studies confirmed the efficacy of TA on significantly reducing ptau (Ser202 and Ser396) in C57Bl/6 mice and increasing expression of p-AKT (Ser473) and p-GSK3β (Ser9) only 4 to 6 h after administration in Wistar rats [41]. In the same study, TA treatment also reduced ptau (Ser396, Thr231) in vitro, and decreased p-PP2A (Tyr307), a protein phosphatase that mediates almost 70% of phosphatase activity, in vitro and in vivo [41].

The cognitive enhancement effects of TA treatment have been well-documented. hTau transgenic mice administered TA for 34 days had significantly reduced escape latencies during the MWM test on day 6, and TA-administered mice spent significantly more time in the correct quadrant during the probe trial [42]. These effects were sustained in mice from 3–4 months of age and in mice 16–18 months of age [42]. In another study, TA treatment also increased the preferential index percentage during the novel object recognition test after short-term treatment (25 days), spent longer times in the correct quadrant during MWM testing, and decreased the amount of error during the passive avoidance test [41].

The overall efficacy that TA exhibits on AD-related biomarkers is still being uncovered; however, it relies on the presence of tau [37]. In a tau knockout model, mice administered TA for 34 days traveled significantly less distance and had decreased protein levels of CDK5 and co-activator p25 than hTau control mice [37]. Both tau knockout mice and hTau mice had reduced protein expression of COX-2, suggesting that TA treatment alters protein expression and improves memory retention in the presence of tau and that the mechanism of tau pathway alterations is independent of its COX inhibitory properties [37].

Inflammation has recently become a major area of focus for neurodegenerative research and Alzheimer’s disease. Many neurodegenerative diseases are accompanied by neuroinflammation as an overarching symptom or possible contributor to the disease. The NLR family pyrin domain-containing 3 (NLRP3) inflammasome is commonly associated with inflammatory diseases, including AD, as Aβ is a known activator [2]. The NLRP3 inflammasome is a critical component of the innate immune system and is composed of NLRP3, associated speck-like protein (ASC), and pro-caspase-1 [48,49]. Daniels et al. (2016) reported that both FFA and MFA inhibited this pathway, in vitro and in vivo, via inhibition of voltage-gated anion channels (VRAC) [2]. Furthermore, MFA treatment prevented Aβ_1–42_-induced memory deficits in rats and memory deficits in 3X TgAD transgenic mice as measured by the novel object recognition (NOR) test [2]. MFA was also effective in decreasing the production of nitric oxide and reducing cytochrome c release from mitochondria induced by Aβ_1–42_ in vitro, and attenuated learning and memory impairment in an Aβ_1–42_-infused AD rat model [1]. MFA treatment also ameliorated AD-related neuroinflammation in 3X TgAD transgenic mice by reducing microglial activation to that of wild-type mice [2]. In agreement with this study, Feng et al. (2020) reported that MFA treatment reduced activated and phagocytic microglia in the dentine gyrus area of the hippocampus in wild-type mice [50]. Another important inflammatory target is nuclear factor kappa B (NF-κB), which is a sequence-specific transcription factor. NF-κB regulates a broad range of genes involved in inflammation, apoptosis, and tumorigenesis. In the presence of inflammatory stimuli, TA downregulates NF-κB signaling [51]. In another study, FFA inhibited the NF-κB signaling pathway at low concentrations [52]. Overall, these results demonstrate that fenamates have clinical significance as AD- and inflammation-targeting drug candidates.

In AD patients, neurotoxic levels of D-serine, the co-agonist for NMDA receptors, have been reported in the hippocampus and is involved in the pathogenesis of AD and neuroinflammation [53]. According to Armagan et al. (2012), MFA treatment protected against the elevation of lipid peroxidation, protein oxidation, and inflammatory targets in the Sprague-Dawley rat brains and may be an effective therapeutic for D-serine-induced neuroinflammation [53].

## 4. Fenamates and Cognitive Impairment

As healthcare has improved, life expectancy has extended, leading to substantial increases in the number of individuals over the age of 65 years old who are at risk of developing cognitive impairment and dementia [54]. Mild cognitive impairment (MCI), a term used for over four decades, describes individuals with a noticeable decline in cognitive abilities that does not interfere with their daily functioning [55]. There are many different etiologies of MCI, including vascular, neurodegenerative, psychiatric, and medical [56,57,58]. Pharmacological interventions are limited and systematic reviews using randomized controlled trials have not investigated a protective effect of dementia medications or NSAIDs [54]. The following section focuses on studies where fenamates have been used to treat non-AD-related cognitive impairment from different etiologies.

### 4.1. Fenamates and Tauopathies

In AD, the characteristic hallmarks are Aβ plaques and neurofibrillary tau tangles (NFTs), and the earliest symptom of AD is cognitive impairment, as mentioned above. Many studies have suggested that the progression of mild cognitive impairment found in AD is most closely associated with NFT formation than Aβ plaque load [43]. Tau aggregation is not unique to AD; in fact, tau aggregation accounts for more than 20 neurological disorders known as tauopathies, which include AD, frontotemporal dementia with parkinsonism-17 (FTD-17), progressive supranuclear palsy (PSP), corticobasal degeneration, chronic traumatic encephalopathy, and Parkinson’s disease (PD) [59]. Moreover, therapeutics that are useful for one tauopathy may be therapeutic in others to combat cognitive decline. In fact, TA has been designated by the FDA and European Medicines Agency (EMA) as an orphan drug for the treatment of PSP, with a clinical trial currently under final approval to evaluate TA in individuals with PSP (NCT04253132).

### 4.2. Fenamates and Chronic Alcohol Exposure

Alcoholism is a chronic disorder, accounting for 5.3% of all deaths worldwide, that is known to be highly correlated with multiple neuropsychiatric diseases and cognitive impairment [30,60]. Several studies have shown that over-consumption of alcohol is a risk factor for dementia via many proposed pathways. Chronic alcohol use and withdrawal may stimulate Toll-like receptors (TLRs) 2 and 4, which could directly lead to microglial activation, neuroinflammation, and neuronal death [30]. Studies have also demonstrated that alcoholic individuals have increased Iba-1 expression, which is a marker of microglial expression [61].

Rajesh et al. (2017) observed that chronic alcohol exposure produced cognitive impairment in zebrafish, evidenced by the inability to retain the memory of a learned task, whereas zebrafish exposed to alcohol and MFA significantly retained memory of the learned task [62]. Furthermore, MFA exposure decreased acetylcholine esterase (AChE) activity in the brain of alcohol-exposed zebrafish [62]. The proposed mechanism for the protective effect was a generation of MFA free radicals during interaction with peroxidase, which was originally reported by Muraoka and Miura (2009) [22].

### 4.3. Fenamates and Ischemic Injury

Cerebral ischemia due to cerebral vessel blockage is a leading cause of death worldwide and neurons are uniquely vulnerable to ischemic injury [63,64]. According to the American Heart Association, stroke is the fifth leading cause of death in the United States [65]. Stroke is typically classified into two categories: ischemic or hemorrhagic [66]. Glutamate-induced excitotoxicity, reperfusion injury due to reactive oxygen species (ROS), neuroinflammation mediated by excessive microglia activation, and impaired axonal regeneration have all been proposed as potential underlying mechanisms of ischemic brain injury [67].

Studies have shown that many fenamates have neuroprotective effects against excitotoxicity-induced cell death [64,68]. MFA and MCFA have been proven to be neuroprotective against glutamate-evoked excitotoxicity in cultured embryonic rat hippocampal neurons, and intracerebroventricular (ICV) administration of MFA for 24 h reduced brain damage in rodents [68]. In another study, treatment with MFA reduced cerebral edema, infarct volume, total ischemic brain damage, and edema, which provided evidence that fenamate NSAIDs may be neuroprotective against ischemic stroke [69].

## 5. Fenamates and Huntington’s Disease

Huntington’s disease (HD) is an autosomal-dominant neurodegenerative disorder caused by the abnormal expansion of the cytosine–adenine–guanine (CAG) repeat in the *IT15* gene located on chromosome 4 [70]. This results in the production of mutant huntingtin (mHtt) protein with a long polyglutamine stretch in the N-terminus region of the Huntingtin protein (Htt) [70,71]. Individuals with greater than 39 CAG repeats develop HD, while those with 36–39 have reduced penetrance [71].

The mean prevalence of HD is estimated at 5.5 cases per 100,000 in the world [72]. Common symptoms are motor dysfunction, psychiatric disturbance, and cognitive deficits including chorea and loss of coordination [71,73]. mHtt causes selective neuronal loss in the striatum (caudate nucleus and putamen) with specific loss of efferent medium spiny neurons (MSNs) and dysfunction in the brain through multiple mechanisms [71,73]. The presence of mutant huntingtin aggregates found initially in the nucleus and later in the cytoplasm and neuronal processes, are considered the hallmark of HD [73].

The most common mouse model of HD is the R6 transgenic model that expresses a truncated form of the human Htt and has been used to examine therapeutic strategies [70]. The human *htt* gene is among many that are regulated by Sp1, which suggests that TA may be an effective treatment to attenuate motor and cognitive deficits for HD patients [74]. In fact, R6/1 mice treated with TA demonstrated improved motor performance in the rotarod test and attenuated cognitive decline, as observed during the novel object recognition (NOR) and passive avoidance tests [70]. Furthermore, TA treatment significantly decreased protein expression of mHtt and SP1 in vivo, and alleviated oxidative stress in PC12 cells [70]. These results suggest that TA treatment may facilitate the clearance of mutant Htt aggregates by SP1 inhibition and activation of the autophagy pathway [70].

## 6. Fenamates and Epilepsy

Epilepsy is a neurological disorder characterized by recurrent unprovoked seizures and is the fourth most common neurological disorder affecting approximately 50–65 million people worldwide [75,76]. Cognitive impairment, mood, and behavior disorders are common comorbidities of epilepsy [77]. Epileptic dementia is a term that was coined in the 19th century based on the idea that epilepsy causes progressive cognitive decline [77]. Currently, epilepsy therapeutics target ion channels or neurotransmitter systems; however, these treatments only provide relief for 60% of patients [76]. The following section reviews how fenamates are known to modulate several ion channels that are implicated in epileptogenesis.

Physiologically, epilepsy is linked to neuronal hyperexcitability [78]. The regulation of excitability is partially controlled by subthreshold, voltage-gated K^+^ currents (M-currents) that are generated by M-type K^+^ channels [78]. The low-threshold gating and slow activation and deactivation of the M-current provide relief from repetitive firing and neuronal excitability [78,79]. According to Peretz et al. (2005), MCFA is a potent and specific opener of KCNQ2/Q3 channels, enhanced M-currents, and reduced evoked action potentials [78].

Voltage-gated sodium channels (VGSC) have also been linked to neuronal disorders such as epilepsy, autism, muscle disease, and pain [80]. The VGSC subtypes Nav1.7 and Nav1.8 have been implicated as targets for pain management [80]. MFA, FFA, and TA inhibited peak sodium currents and significantly affected the inactivation processes of hNav1.7 and hNav1.8 with I-V curves left-shifted to the hyperpolarized direction. These findings may contribute to the well-known analgesic effects of these fenamates [80]. In another study, FFA inhibited VGSC currents in hippocampal pyramidal neurons by slowing down the inactivation process of the sodium current and shifting the inactivation curve toward more hyperpolarized potentials [81].

## 7. Overview of Alternative Drugs

**Figure 2:**
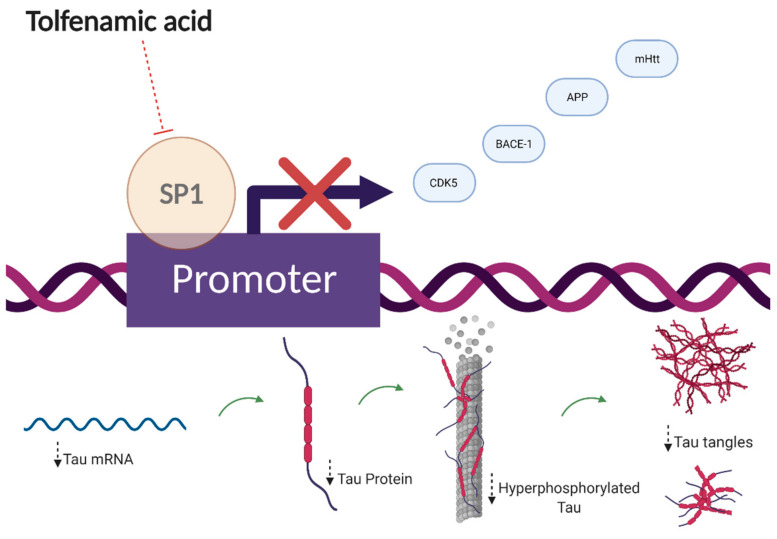
Proposed transcriptional mechanism of action for tolfenamic acid. Tolfenamic acid inhibits specificity protein 1 (SP1)-DNA binding, which leads to decreased expression of amyloid precursor protein (APP), mutant huntingtin protein (mHtt), β-secretase-1 (BACE-1), and cyclin-dependent kinase-5 (CDK5). The bottom half of the illustration shows the impact of tolfenamic acid on tau tangles via SP1 inhibition.

**Figure 3:**
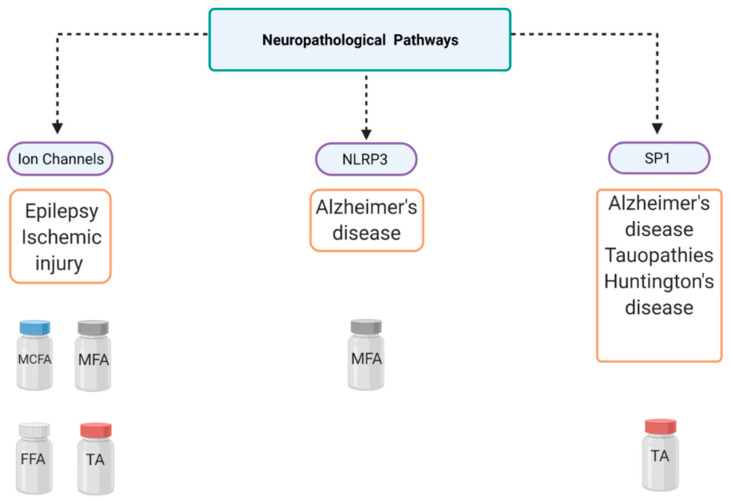
Mechanistic overview of fenamate neuropharmacology. This figure illustrates several diseases that can be targeted using fenamates and their three major pathways of interest. Tolfenamic acid (TA), mefenamic acid (MFA), meclofenamic acid (MCFA), flufenamic acid (FFA), NLR family pyrin domain-containing 3 (NLRP3), specificity protein 1 (SP1).

In this review, we have discussed three key neurodegenerative pathways modulated by fenamates (Figure 2 and Figure 3). It is also imperative to acknowledge the originality of each major mechanism and other drugs that may provide similar effects. The following section overviews the currently approved or potentially therapeutic drugs that use similar molecular mechanisms as stated above.

In the past, NSAIDs were extensively studied for their possible role as cancer therapeutics. Chronic inflammation has been linked widely to carcinogenesis, and epidemiological studies have indicated the chemoprotective and chemopreventive properties of NSAIDs [82]. Fenamate NSAIDs are known to modulate several pathways and exhibit anti-tumor activities in models for several cancers [83,84,85,86]. In fact, The SP1 hypothesis for AD intervention originated from cancer research as SP1 is upregulated in various types of cancer [87]. Although TA is unique among NSAIDs for its’ ability to target SP1, TA is not the only known therapeutic able to modulate SP1 binding and activity. Mithramycin (MTM) is an antineoplastic antibiotic that has been proven to bind to GC-rich DNA sequences and thus interferes with transcription factors that bind to GC-rich DNA regions, such as SP1 [87,88,89]. It has also been suggested that MTM can selectively inhibit SP1 but the mechanism is not well understood [87]. MTM treatment reduced cerebral Aβ levels and plaque burden, inhibited APP processing, alleviated tau hyperphosphorylation, and inhibited phosphorylated CDK5 and GSK3β pathways in APPswe/PS1dE9 mice [90]. MTM treatment also upregulated synaptic plasticity gene expression in an AD model in vitro, and prolonged survival and improved motor performance in an HD mouse model [89,91]. Collectively, these reports support the SP1 hypothesis for neurodegenerative disease intervention.

Furthermore, the role of COX inhibition in neurodegenerative and neuroinflammatory treatment or prevention is pertinent to mention. COX-2 is expressed under basal conditions in neuronal regions that overlap with AD pathogenesis [92]. It has even been suggested that the overexpression of neuronal COX-2 activity may disrupt normal neuronal function [92]. Due to the evidence linking COX-2 dysregulation to neurodegenerative diseases, NSAIDs have been implicated for their potential therapeutic effects in neurodegenerative disease states, particularly AD. In APP-PS1 mice, administration with ibuprofen during early adulthood prevented memory deficits [92]. However, the correlation of COX inhibition and the decreased occurrence or progression of neurodegenerative diseases has been debated because, although epidemiological data have shown that populations with long histories of NSAID use were at lower risk of AD, clinical studies have failed to achieve significant effects in the treatment of AD [93]. These findings may suggest the idea that NSAIDs may be useful in neurodegeneration prevention but are less useful for treatment; however, there has been no conclusive data proving their efficacy in prevention in humans.

Alzheimer’s disease is the only top-10 cause of death without a disease-modifying treatment approved by the FDA. Several recent anti-amyloid therapies have failed in clinical trials, which has prompted researchers to explore alternative pathways to target [94]. Targeting neuroinflammation is a recent approach that has gained more attention due to the high failure rate of previous drug candidates [94]. The NLRP3 inflammasome is a well-known driver of tau pathology and there are several proven direct and indirect inhibitors of the NLRP3 inflammasome that have been identified [48,95,96]. However, inhibition of VRAC by MFA is a novel approach to NLRP3 inhibition, and researchers have suggested that it is important to consider indirect therapeutic methods as targeting the inflammasome itself may result in peripheral complications [97].

Anti-seizure drugs (ASDs) are the predominant form of treatment for symptomatic relief for patients living with epilepsy [98]. ASDs interact with a variety of cellular targets including voltage-gated ion channels, GABA_A_ receptors, GABA transporter 1 (GAT), synaptic vesicle proteins, ionotropic glutamate receptors, and several other targets of interest [98]. Currently, there are about 30 ASDs on the market [98]. While targeting VGSCs is not a novel mechanism, to our knowledge, there are no current commonly used drugs that target KCNQ2/Q3 channels [99]. Retigabine was discovered in the 1980s as a potent opener of channels formed by KCNQ2 and KCNQ3 subunits and was used to treat neonatal epileptic encephalopathy and, unfortunately, it was discontinued in 2017 due to pigmentary changes induced in skin, mucosae, and eyes [79,100]. For acute ischemic stroke, glutamate-induced excitotoxicity is a well-known contributor to ischemic neuronal death and has been targeted indirectly by NMDA receptor antagonists [101]. Thus far, there are no FDA-approved drugs for cerebral ischemia that target glutamate excitotoxicity, to our knowledge, as several have failed in randomized controlled clinical trials in humans [101]. There have been recent novel advances to combat glutamate-excitotoxicity, such as introducing glutamate scavengers or “grabbers”, which further reiterates the idea that glutamate excitotoxicity remains an important target for cerebral ischemia [102].

## 8. Discussion and Conclusions

Altogether, we have presented mounting evidence of several non-canonical disease pathways, from Alzheimer’s disease to epilepsy, that are modulated by various fenamates (Figure 2 and Figure 3), and there are several significant conclusions to be mentioned in terms of this review. First, it is important to consider fenamates in neuropathological drug development as modulating several pathways may provide greater protective effects than molecules that have single targets, which may allow them to be efficacious at lower doses [2]. The main benefit may be in lowering inflammation as well as other disease-specific targets. Fenamates seem to act directly on their targets such as enzymes and ion channels, as well as indirectly by modulating transcription factors and thus impacting disease-specific gene expression. Interestingly, the ability of certain fenamates to activate proteasome-dependent degradation of SP1, and inhibit NLRP3 and certain ion channels is unique among NSAIDs, which may be indicative of a chemical class effect instead of a therapeutic class effect (NSAID) [2,34,103]. Drug repurposing offers many benefits as it may expedite the drug discovery process by shortening the high-throughput screening and clinical trial phases and provide proven safe alternatives to existing treatments. This is particularly important as CNS drugs have high failure rates and tend to have poorly understood pathways, and often patients have several co-morbidities. Furthermore, it is important to consider the adverse reactions that have been well documented due to long-term NSAID use and how the length of therapy and the therapeutic dose required may determine the safety of these small molecules for age-related disorders. In fact, fenamates could serve as a scaffold in future therapeutic drug design for neurodegenerative diseases. Finally, these data suggest the effect that fenamates have is polyvalent and substantiates the hypothesis that some therapeutic effects are independent of the COX pathway. In the future, an exploratory pathway analysis and a comprehensive safety profile for each drug could provide insight into drug mechanisms of action and possible adverse effects specific to fenamates.

## Data Availability

Data sharing not applicable.

## Abbreviations

NSAIDS	Nonsteroidal anti-inflammatory drugs
COX	Cyclooxygenases
PG	Prostaglandin
IC_50_	Inhibitory concentration
MFA	Mefenamic acid
TA	Tolfenamic acid
MCFA	Meclofenamic acid
FFA	Flufenamic acid
PHGS	Prostaglandin H synthase
AD	Alzheimer’s disease
MRI	Magnetic resonance imaging
CSF	Cerebral spinal fluid
LOAD	Late-onset Alzheimer’s disease
EOAD	Early-onset Alzheimer’s disease
FDA	Federal Drug Administration
NMDA	N-Methyl-D-aspartic acid
APP	Amyloid precursor protein
BACE-1	β-Secretase-1
SP1	Specificity protein 1
MWM	Morris water maze
MAPT	Microtubule-associated protein tau
CDK5	Cyclin-dependent kinase-5
siRNA	small interfering RNA
GSK3β	Glycogen synthase kinase-3 beta
ptau	Phosphotau
hTau	Human tau
NLRP3	NLR family pyrin domain-containing 3
NF-κB	Nuclear factor kappa B
MCI	Mild cognitive impairment
NFT	Neurofibrillary tau tangles
FTD-17	Frontotemporal dementia with parkinsonism-17
PSP	Progressive supranuclear palsy
PD	Parkinson’s disease
EMA	European Medicines Agency
TLRs	Toll-like receptors
AChE	Acetylcholine esterase
ROS	Reactive oxygen species
ICV	Intracerebroventricular
HD	Huntington’s disease
CAG	Cytosine–adenine–guanine
mHtt	Mutant huntingtin
Htt	Huntingtin protein
MSNs	Medium spiny neurons
ASC	Associated speck-like protein
VRAC	Voltage-gated anion channels
NOR	Novel object recognition
VGSC	Voltage-gated sodium channels
MTM	Mithramycin
ASD	Anti-seizure drugs
GAT	GABA transporter 1
